# Mapping the Burden of Tegumentary Leishmaniasis in South America: A Systematic Review and Meta‐Analysis

**DOI:** 10.1111/tmi.70035

**Published:** 2025-09-09

**Authors:** Sérgio Caldas, Job Alves de Souza Filho, Janete Soares Coelho Santos, Felipe Campos de Melo Iani, Cristiane Faria de Oliveira Scarponi

**Affiliations:** ^1^ Diretoria de Pesquisa e Desenvolvimento Fundação Ezequiel Dias Minas Gerais Brazil

**Keywords:** cutaneous leishmaniasis, diagnosis, *Leishmania*, prevalence, South America, zoonosis

## Abstract

**Introduction:**

In recent years, the global burden of tegumentary leishmaniasis (TL) has significantly increased in the Americas.

**Objective:**

To estimate the prevalence of TL in South America based on publications from the past 13 years.

**Methods:**

Three databases were searched, and articles were selected based on inclusion criteria and methodological relevance. The random effects model was used to calculate pooled prevalence, and heterogeneity was assessed with the *I* ^2^ statistic.

**Results:**

Out of the 225 articles initially identified, six studies remained eligible for review and meta‐analysis. No publication bias was found. TL prevalence in South America ranged from 0.2% to 87.7%, showing significant heterogeneity (*I* ^2^ = 99.83%, *p <* 0.001). Colombia and Ecuador had the highest prevalence rates (> 76.6%). The pooled prevalence in the general population was 0.4% (95% CI: 0.1–1.0), and 81.8% (95% CI: 75.4–87.4) in symptomatic patients attending health units.

**Conclusions:**

TL prevalence varied widely across countries, reflecting national epidemiological disparities. High positivity rates in suspicious samples and molecular detection in samples confirmed by microscopy underscore the importance of accurate diagnosis and clinical expertise. This meta‐analysis emphasises the need for tailored health policies and accessible laboratory diagnoses to guide disease control strategies.

## 1 Introduction

Tegumentary leishmaniasis (TL) is one of the most neglected zoonotic diseases, posing a significant health threat due to the complex human‐animal‐environment interface. The disease has a high capacity for skin and mucous membrane deformation, leading to substantial psychosocial impact. It is caused by various species of protozoa from the *Leishmania* genus, transmitted during the blood meal of infected female sandflies. TL is present in more than 85 countries in tropical and subtropical regions, with a worldwide incidence of 0.7 to 1.2 million confirmed cases per year. The contact with the parasite does not confer immunity to the patient [1, 2]. In Latin America, this type of infection has been reported in at least 17 countries. In 2019, the Pan American Health Organization recorded 41,617 cases, with over 35% occurring in Brazil [3].

In some individuals, the infection may remain asymptomatic or subclinical [4]. The clinical manifestations of the disease depend on the *Leishmania* species, the vector responsible for transmission, and the host's immune response [2, 3]. Single or multiple lesions on the skin, described as shallow ulcers with raised edges and a grainy bottom, characterise cutaneous leishmaniasis (CL). These lesions are typically painless and often occur on the face, arms, and legs. They can scar spontaneously. Mucocutaneous leishmaniasis (MCL), on the other hand, is a secondary lesion that destructively affects the mucous membranes of the oropharynx, with involvement of the cartilaginous septum, and can progress chronically to disfiguring lesions [1, 5]. CL is the main clinical presentation, accounting for 90% of cases, and is distributed worldwide, predominantly in tropical and subtropical regions. In the Americas, there are TL cases reported from the south of the United States to the north of Argentina [1].

A presumptive diagnosis of TL can be based on clinical and epidemiological criteria. However, the lesions can be confused with other dermatoses, whether of infectious origin or not, requiring their association with differential diagnosis. The gold standard for laboratory diagnostic confirmation consists of microscopic identification of *Leishmania* amastigote forms in tissue or promastigotes in culture media. Serological and molecular tests also play a crucial role in confirming the diagnosis [6, 7, 8]. TL is a treatable disease; however, drugs do not eliminate the parasite from the body but improve prognosis and reduce the chances of mutilation. The risk of relapse is high if the individual is immunosuppressed. Early diagnosis and immediate treatment help reduce the prevalence and transmission of this protozoan [1, 9].

A review of recent studies allows us to identify consistent epidemiological patterns to improve the prevention and control of leishmaniasis. Thus, the aim of this meta‐analysis was to estimate the pooled prevalence of human TL in South America. The consolidated information is expected to assist public officials in decision‐making, optimise available resources, and enhance the effectiveness of health surveillance efforts.

## 2 Methods

### 2.1 Sources of Information and Search Strategy

This study was conducted following the guidelines of the “Preferred Reporting Items for Systematic Reviews and Meta‐Analyses” (PRISMA). Its protocol was registered (Available at: http://www.crd.york.ac.uk/PROSPERO/display_record.php?ID=CRD42021589243) in the PROSPERO platform [10]. The bibliographic searches were conducted to identify studies published between 2010 and 2023. MeSH terms or keywords (“*Leishmania*”, “human”, “infection”, “prevalence”, “frequency” and “country”) associated with Boolean connectives (OR/AND) were used to compose a comprehensive search algorithm. Each country in the South American region was searched individually: Argentina, Bolivia, Brazil, Chile, Colombia, Ecuador, Guyana, Paraguay, Peru, Suriname, Uruguay, and Venezuela. The full search sequence is available in Table 1. The most recent search for scientific articles was conducted in the EmBase, SciElo, and PubMed databases on March 31, 2024.

**TABLE 1:** Search strategy into electronic databases for systematic review.

Database	Date and time accessed	Advanced search	Nr of records
PubMed	March 30, 2024; 10:46 p.m.	(“human”) [All fields] AND (“leishmania” OR “leishmaniasis”) [All fields] AND (“prevalence” OR “frequency”) [All fields] AND “Country*” [All fields] AND “2012–2022” [Filter: results by years]	78
EmBase	March 30, 2024; 15:00 p.m.	(“human”) [All fields] AND (“leishmania” OR “leishmaniasis”) [All fields] AND (“prevalence” OR “frequency”) ([Title or Abstract]) AND “Country*” [Title or Abstract] AND “2012–2022” [Publication years (including)]	141
SciElo	March 31, 2024, 12:05 p.m.	(“human”) [All indexes] AND (“leishmania” OR “leishmaniasis”) [All indexes] AND (“prevalence” OR “frequency”) [All indexes] AND “Country*” [All indexes] AND “2012–2022” [Filter: Publication years]	6

### 2.2 Study Selection

Bibliographic references found in the databases were compiled into a spreadsheet, and any duplicates were removed. Subsequently, three reviewers independently screened the abstracts and full texts, selecting studies according to inclusion and eligibility criteria. Any disagreements were resolved by consensus. Duplicate records, studies that did not meet the predefined requirements, and articles with inaccessible full texts were reasons for exclusion. Only studies fulfilling all inclusion and eligibility criteria were retained for qualitative synthesis and meta‐analysis.

### 2.3 Inclusion and Eligibility Criteria

The inclusion criteria were: *Population*—humans residing in South America; *Exposure*—to *Leishmania* spp.; *Comparison*—of laboratory diagnostic results for tegumentary leishmaniasis; *Outcome*—the quantitative measure of infection cases or prevalence; *Temporality*—published articles between 2010 and 2023; and *Study design*—observational, cross‐sectional, case–control, or cohort. There were no restrictions regarding the age, sex, or race of the participants, nor the language of the publication. The following laboratory markers were considered valid for confirming the diagnosis of *Leishmania* infection: positivity in the indirect immunofluorescence assay (IFA), *Leishmania* skin test (LST), Enzyme‐Linked Immunosorbent Assay (ELISA), detection of the protozoan by microscopy, or amplification of genomic regions by reaction in polymerase chain reaction (PCR).

The methodological quality of the preselected studies was evaluated using the modified Joanna Briggs Institute (JBI) Checklist [11]. This instrument comprises 10 questions designed to evaluate potential sources of bias, and each answer scored as 0 for “No/Unclear” and 1 for “Yes.” The quality of each study was obtained by adding the individual scores, and the studies were subsequently classified as low risk of bias (8 to 10 points) or moderate risk of bias (5 to 7 points), with those with high risk of bias (final score less than 5 points) being excluded.

### 2.4 Data Extraction

The raw data from the included studies in the review and meta‐analysis were independently extracted by two reviewers and subsequently verified for accuracy by a third reviewer. The data included: the year of publication, authors, country studied, period of data collection, study design, sample size, number of cases identified with leishmaniasis, and other relevant information.

### 2.5 Summary of Results and Statistical Analysis

The individual prevalence rate was calculated by dividing the absolute number of cases identified with TL by the total number of patients tested per study. The extracted data were analysed using the “metaprop” and “meta” commands in Stata software version 11.0 (Stata Corp., College Station, TX, USA), with *p* values < 0.05 considered significant [12]. Due to the expected heterogeneity between the selected studies, a random effect meta‐analysis was used with double arcsine transformation to calculate the pooled prevalence with a 95% confidence interval (CI) [13]. The calculation of the *I* ^2^ statistic established the percentage of heterogeneity (considered high if *I* ^2^ > 75%) between studies, and possible sources of variation were investigated through subgroup and meta‐regression analyses. Furthermore, publication bias was assessed using funnel plot inspection, Begg and Mazumdar's rank correlation test and Egger's linear regression test [14].

## 3 Results

**FIGURE 1:**
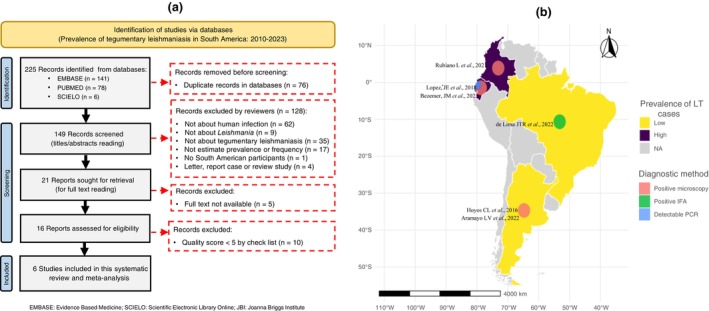
A systematic review on the prevalence of tegumentary leishmaniasis in South American countries: (A) Flowchart of the selective steps for inclusion and exclusion of studies; (B) Geographic distribution of studies by country. EMBASE, Evidence Based Medicine; JBI, Joanna Briggs Institute; SCIELO, Scientific Electronic Library Online.

Through a comprehensive search of three databases, 225 articles related to the prevalence of TL in South America were identified. Of these, 219 articles were removed for not meeting the predetermined eligibility and inclusion requirements. The steps involved in selecting relevant studies are illustrated in Figure 1A. Basic information extracted from the six articles included in this review and meta‐analysis is described in Table 2.

**TABLE 2:** Main data compiled from six studies that address tegumentary leishmaniasis in South America (published articles between 2010 and 2023).

Publication year	Study authors	Country	Geographic location	Collection data	Design study	Sample size	Population type	Diagnostic method	Presence of *Leishmania* (microscopy)	Positive IgG *Leishmania* (IFA)	Detectable *Leishmania* (PCR)	Tegumentary leishmaniasis cases (Total)	Quality level
2016	Hoyos CL et al.	Argentina	Hipolito Yrigoyen	2009	Cross‐sectional	10,363	General	Microscopy, LST, ELISA	18			18	8
2017	Lima JTR et al.	Brazil	Mato Grosso	2011	Cross‐sectional	470	General	IFA		2		2	6
2018	Lopez, JE et al.	Ecuador	Tena‐Napo	2012–2013	Retrospective	220	CL‐suspected patient	Microscopy	180			180	8
2021	Rubiano L et al.	Colombia	Tumaco	2015–2016	Prospective	122	CL‐suspected patient	Microscopy	107			107	8
2022	Aramayo LV et al.	Argentina	Colonia Santa Rosa	1985–2019	Retrospective	16,000	General	Microscopy, LST	120			120	7
2023	Bezemer, JM et al.	Ecuador	Pichincha, Napo, Pastaza, and Morona Santiago	2019–2021	Cross‐sectional	320	CL‐suspected patient	Microscopy, PCR	110		135	245	8

Abbreviations: CL, Cutaneous leishmaniasis; ELISA, Enzyme‐Linked Immunosorbent Assay; IFA, Indirect immunofluorescence assay; LST, *Leishmania* skin test; PCR, polymerase chain reaction.

Across the rescued studies, the most commonly identified sources of methodological bias were inadequate reporting of participant characteristics and the lack of explicit sample size calculations. The six articles included in the review and meta‐analysis demonstrated a mean quality score of 7.5 (range: 6–8), indicating an overall moderate‐to‐high level of methodological rigour, even with different study designs [5, 15, 16, 17, 18, 19]. Furthermore, was no evidence of publication bias was revealed by Begg's test (*z* = 1.69, *p* = 0.091) and Egger's test (regression coefficient = −0.09, *p* < 0.131).

In total, these studies included 27,495 samples collected between 1985 and 2021, with 672 laboratory‐confirmed cases of TL. The LC form was reported in all six selected studies; MCL was described in only one study with 13 confirmed cases [15]. Figure 1B shows studies in four of the 12 South American countries, published between 2016 and 2023, involving two types of populations (general and symptomatic patients treated in health units).

**FIGURE 2:**
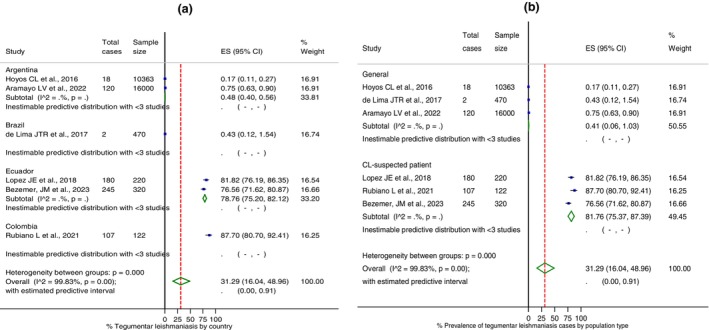
Forest plot of the % prevalence of tegumentary leishmaniasis in South American countries (published articles between 2010 and 2023): (A) by country; (B) by population type. Output generated by Stata procedure metaprop. Weights were calculated from random effects meta‐analysis. CI, Confidence interval; ES, effect study (% frequency). Predictive interval expressed in proportion.

Individual and combined TL prevalence rates from studies in this region were estimated by meta‐analysis, and their results are shown in Figure 2A. The combined prevalence of leishmaniasis across four South American countries was 31.3% (95% CI: 16.0–49.0). Argentina and Brazil presented studies with the lowest rates of leishmaniasis, 0.2% and 0.4%, respectively [15, 16, 19]. On the other hand, in the Andean region, Ecuador reported rates ranging from 76.6% to 81.8%, while Colombia recorded the highest prevalence in South America at 87.7%. Substantial differences in individual prevalence rates were evident and could be confirmed by the statistical test (*I* ^2^ = 99.83%, *p <* 0.001).

Additional subgroup analyses were conducted to investigate the high heterogeneity between studies, as shown in Table 3. Given the observation that one of the included studies reported data collected over an extended period (1985–2019), its potential impact on the pooled prevalence rate of tegumentary leishmaniasis was carefully assessed through subgroup analysis and meta‐regression [15]. According to the meta‐regression results, this effect was not statistically significant. However, the meta‐regression results (Table 4) revealed a strong association only with the country of the study (*p* = 0.012). This variable partially accounted for the high heterogeneity in TL occurrence across the South American region (adjusted *R* ^2^ = 81.06%).

**TABLE 3:** Subgroup analyses of studies on tegumentary leishmaniasis in South America (published articles between 2016 and 2023).

Subgroups	Number of studies	% TL cases occurrence	95% Confidence interval	*I* ^2^ (%)	*p* ‐value (intra sub‐group)	*p* ‐value (between sub‐groups)
Country						
Argentina	2	0.5	0.4–0.6			0.00*
Brazil	1	0.4	0.1–1.5			
Colombia	1	87.7	80.7–92.4			
Ecuador	2	78.8	75.2–82.1			
Data collection period						
≤ 2012	2	0.1	0.1–0.2			0.00*
2012–2018	2	84.0	79.9–87.8			
> 2018	1	76.6	71.62–80.87			
EP (1985–2019)	1	0.7	0.6–0.9			
Population type						
General injuries	3	0.4	0.1–1.0			0.00*
TL suspected injuries	3	81.8	75.4–87.4			
Diagnostic method						
IFA	1	0.4	0.1–1.5			0.00*
Microscopy, LST, ELISA	1	0.2	0.1–0.3			
Microscopy	3	52.2	0.0–100.0			
Microscopy, PCR	1	76.6	71.6–80.9			
Quality level						
Moderate	2	0.7	0.6–0.8			0.04*
High	4	57.5	1.34–100.0	99.90	0.00	

*Note*: Output generated by Stata procedure *metaprop* by random model effects.

Abbreviations: ELISA, Enzyme‐Linked Immunosorbent Assay; EP, Extended period; IFA, Indirect immunofluorescence assay; LTS, *Leishmania* skin test; TL, Tegumentary leishmaniasis.

* Considered statistically significant *p*‐value ≤ 0.05.

**TABLE 4:** Summary of univariate meta‐regressions for tegumentary leishmaniasis in South America (publication from 2010 to 2023).

Groups	Coefficient β	*p* ‐value	95% Coefficient interval
Publication year	−0.052	0.769	−0.057 to 0.468
Country	0.292	0.012*	0.108 to 0.476
Collection period	0.164	0396	−0.315 to 0.643
Population type	0.239	0.574	−0.846 to 1.325
Sample size	−0.000	0.130	−0.0001 to 0.0002
Quality criteria score	0.346	0.166	−0.222 to 0.913

*Note*: Output generated by StataTM procedure metaprop.

* Considered statistically significant *p* ‐value ≤ 0.05.

**FIGURE 3:**
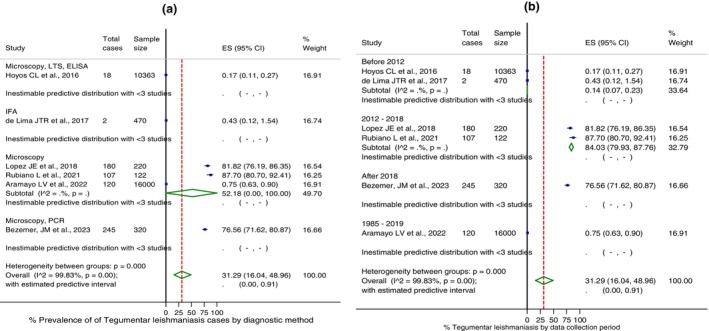
Forest plot of % diagnosis for tegumentary leishmaniasis in South American countries (articles published between 2010 and 2023): (A) by diagnostic method; (B) by data collection period. Output generated by Stata procedure metaprop. Weights were calculated from random effects meta‐analysis. CI, Confidence interval; ES, effect study (% frequency). Predictive interval expressed in proportion.

The prevalence rates of TL were higher in patients with clinical suspicion who sought medical care (81.8%) compared to the general population (0.4%), according to studies carried out (Figure 2B). Regarding the different combinations of diagnostic methods (Figure 3A), the highest rates of parasitological positivity were diagnosed using microscopy and PCR techniques [18]. Considering exclusively the positive microscopy, the identification rate of *Leishmania* in lesions with clinical suspicion was 0.5% (95% CI: 0.4–0.6) in the general population, and 69.7% (95% CI: 32.2–96.2) in patients in healthcare facilities (data not shown). The molecular detection rate of the protozoan was 42.2% (95% CI: 36.9–47.7) in samples previously positive by direct microscopy, as estimated based on a single study [18]. This was particularly evident for biological samples collected after 2012 (Figure 3B).

## 4 Discussion

This meta‐analysis underscores the significant presence of leishmaniasis in two South American countries, Colombia and Ecuador. It also reveals the limited number of studies conducted in the region over the past 13 years. This gap in the literature emphasises the need to intensify efforts in designing future epidemiological research and developing effective strategies to control the disease. Therefore, strengthening international partnerships is essential for improving the management and development of health services in the region's countries.

The pooled prevalence of TL (31.3%) across four South American countries was significantly higher than the 12.0% reported in nine Middle Eastern countries [20]. However, both prevalences are likely underestimated due to misdiagnosis and inconsistent reporting guidelines [3]. Such findings align with epidemiological evidence documenting endemic foci from the southern United States to northern Argentina [3, 15]. Globally, TL exhibits a broad geographical distribution, remaining endemic in the Americas, Europe, Africa, and Asia, where it disproportionately affects low‐income populations [1, 3]. The prevalence estimated in this meta‐analysis is more consistent with the 20.4% reported in a recent meta‐analysis, which included 19 studies conducted in Ethiopia [2]. This highlights the widespread nature of this neglected infectious disease, which predominantly affects vulnerable individuals with limited access to public services.

The prevalence rates reported in the six studies are clustered at two extremes, with values below 1.0% or above 70.0%. A stratification of studies into subgroups was necessary to identify potential factors contributing to the high statistical heterogeneity (*I* ^2^ = 99.83%). According to these findings, the polarisation of these rates is independent of whether categorised by country or population type, a pattern consistent with the results of African meta‐analysis [21]. Epidemiological studies revealed low occurrences of this type of leishmaniasis in Argentina and Brazil, in sharp contrast to extremely high rates in underdeveloped countries, such as Ecuador and Colombia. However, in absolute numbers reported in the Americas from 2001 to 2020, Brazil ranks first with 21,535 cases, followed by Colombia with 10,183 and Peru with 7038 [20]. National and local evidence shows distinct transmission patterns between countries, likely due to differences in the organisation of their healthcare services. Another plausible explanation would be the recruitment of study participants (convenience samples or representative of the general population), associated with variation in sample size (< or > 500 individuals).

Studies focusing on patients with lesions suspected to be caused by TL typically show higher laboratory diagnosis positivity rates, especially in endemic countries. Selecting a specific population with existing symptoms increases the likelihood of detecting the parasite in biological samples [19, 22]. This has been confirmed by previous studies conducted on symptomatic patients in Colombia and Ecuador [5, 17, 18]. In contrast, detection rates significantly decrease when testing the general population, as the target group expands to include asymptomatic individuals and those with subclinical lesions unrelated to leishmaniasis, as shown in several studies [2, 15, 16, 19, 21].

Furthermore, *Leishmania* infections are closely associated with poverty, along with environmental and climatic factors that shape the epidemiology of the parasite [3, 21]. Several social factors, including variations in economic activities, land use practices, demographic characteristics, and the effectiveness of vector control and diagnostic efforts, may contribute to the disparities between countries [15, 16, 18, 21]. Another potential factor contributing to the high rates is the inadequate implementation of epidemiological surveillance and control programmes. This issue is pronounced in countries that are still in the process of implementing laboratory diagnosis and local treatment for TL [3].

A crucial factor for epidemiological surveillance is the performance of laboratory diagnoses. Methods for confirming the disease vary regionally, causing differences in TL detection rates and directly influencing prevalence rates [16, 17, 18]. Lima et al. highlight that techniques commonly used for the serological diagnosis of leishmaniasis have low reliability and accuracy [19]. Microscopy, a fast and low‐cost technique, is still used due to its high specificity, despite its sensitivity (15%–30%) in infections with a low density of protozoa, especially if related to species circulating in the Americas [4]. An obstacle that can be overcome with molecular diagnostics or a combination of methods [9, 23].

Molecular diagnosis has gained increasing importance due to its high sensitivity and specificity, approaching 100%, despite the associated costs. Studies indicate that PCR, particularly real‐time PCR, is more sensitive than microscopy, as it can detect even minimal amounts of protozoan DNA, including in samples with low parasite loads [5, 8, 24, 25]. The integration of PCR with other diagnostic methods can enhance sensitivity by more than 80%, making it a highly recommended approach for suspected cases of CL and MCL [9]. These methods are particularly valuable for post‐treatment patients, where the parasite burden may be too low for detection by microscopy.

In this review, only one of the studies performed parasite diagnosis by microscopy in parallel with PCR, allowing the infecting species to be identified in 73% of positive samples [18]. Given the diversity of *Leishmania* species and clinical manifestations, it is important to identify the type of parasite involved [7, 8]. Despite technological advances, there is still difficulty in accessing advanced diagnostic techniques. A challenge for financially disadvantaged regions, where leishmaniasis is hyperendemic and resources are limited. Additionally, standardisation of the PCR technique is necessary to enable direct comparisons between studies and to get more accurate estimates [7].

Other studies have provided information on the epidemiology of TL in regions such as the Amazon in Brazil and the Pacific in Ecuador, findings that corroborate previous studies [16, 18]. In the Americas, most of the 22 *Leishmania* species pathogenic to humans have been identified, with each causing one or two distinct dermatological forms [18, 24]. In the Amazon region, *L. (V.) panamensis, L. (V.) guyanensis*, and *L. (L.) amazonensis* are among the three most frequently reported species. These species have also been associated with isolated cases of MCL, highlighting the complexity and diversity of disease transmission in this region [3, 26]. The distribution of these species is strongly influenced by geography and the interaction between the parasite, animal reservoirs, and vectors.

Molecular diagnostics are crucial for the accurate identification of Leishmania species. Due to their high sensitivity and specificity, *L. guyanensis* , *L. braziliensis* , and *L. lainsoni* were identified by *q*PCR in 73% of confirmed cases of CL in the North Pacific and Central Amazon ecoregions [18, 22, 24, 27]. The significance of these laboratory methods was highlighted in 2022, when 86.2% of all CL cases were confirmed using molecular techniques, reflecting a 7% improvement in diagnostic accuracy compared to the previous year [3]. Adequate identification of *Leishmania* vectors reinforces the need for more accurate diagnoses. Understanding the interactions between vectors, reservoirs, and parasites is crucial for effective disease control.

It is an endemic disease occurring mainly in tropical and subtropical regions, with several species of *Leishmania* and vectors involved. In the Americas, around 54 species of vectors have been identified as potentially involved in transmitting this parasitic infection [3]. Aramayo et al. investigated the presence of leishmaniasis and sand flies in Colonia Santa Rosa, located in northern Argentina, highlighting the persistent transmission of TL over the years. A key finding was that 10.83% of the patients presented with MCL, the more severe clinical form of the disease. *Nyssomyia neivai* was identified as the predominant sand fly species in the peripheral neighbourhoods, accounting for 95% of all specimens collected. Other species were also detected in lower proportions, including *Migonemyia migonei* (1.9%), the c*ortelezzii* complex (1.3%), and *Evandromyia sallesi* [15]. *N. neivai* is considered the primary vector responsible for the endemic transmission of *Leishmania (Viannia) braziliensis* in Argentina. This parasite is recognised as the main etiological agent of this form of leishmaniasis in the New World [15, 27]. *N. neivai* is typically more abundant along the edges of primary and secondary forest vegetation, as well as in areas modified by human activity [15].

The accurate identification of vectors and the understanding of their dynamics are crucial for the development of effective control strategies. The high prevalence of CML in peri‐urban areas highlights the link between socioeconomic factors and disease dynamics. In Mato Grosso, despite the presence of *Lutzomyia longipalpis* and *Lutzomyia flaviscutellata*, *Leishmania* infection is minimally spread among the Tapirapé indigenous people. These vectors may be influenced by local habits and environmental factors, mainly in wild or rural areas, after activities such as cleaning, harvesting, hunting, or fishing reduce biological diversity and eliminate non‐reservoir hosts. Anthropogenic changes increase the likelihood of vector‐reservoir contact and, consequently, the risk of infection transmission to humans [15].

A limitation of this review is the lack of research from over half of South American countries, where TL is likely underdiagnosed and underreported, hindering regional prevalence estimates. Additionally, the findings should be interpreted with caution due to significant heterogeneity between studies. A strength was segregating the population to obtain a more accurate prevalence rate, revealing a lower prevalence in community‐based studies compared to those focusing on symptomatic patients. This review also highlights gaps in leishmaniasis epidemiology and control, emphasising the need for a better understanding of vectors and reservoirs. Future research should focus on developing sensitive, accessible diagnostic methods and improving our understanding of factors influencing TL epidemiology.

## 5 Conclusion

TL prevalence varied widely across South American countries, reflecting regional epidemiological disparities. High positivity rates in suspicious samples and molecular detection in microscopy‐confirmed samples highlight the importance of accurate diagnosis and clinical expertise.

## Ethics Statement

The authors have nothing to report.

## Conflicts of Interest

The authors declare no conflicts of interest.

## Data Availability

The datasets generated and/or analyses in the current study are available from the corresponding author upon reasonable request.
